# Tricuspid Annuloplasty: Transcatheter Approaches

**DOI:** 10.1007/s11886-021-01570-8

**Published:** 2021-08-19

**Authors:** Martin Arnold, Julia Haug, Melanie Landendinger

**Affiliations:** Department of Cardiology, University Hospital Erlangen, Friedrich-Alexander-Universität Erlangen-Nuremberg, Ulmenweg 18, 91054 Erlangen, Germany

**Keywords:** Tricuspid regurgitation, Transcatheter tricuspid annuloplasty, Interventional valve therapy, TTVR

## Abstract

**Purpose of Review:**

New transcatheter techniques to perform tricuspid annuloplasty are evolving and are introduced into the clinical routine. Yet, clinical experience is limited.

**Recent Findings:**

Currently, 3 different techniques for tricuspid annuloplasty have been used in larger clinical cohorts. They can be divided into direct annuloplasty techniques and suture plication techniques. The largest clinical evidence is related to direct annuloplasty techniques. It has been shown that annular dimensions can be effectively reduced. This translates into an improvement of the degree of tricuspid regurgitation and improvement of clinical symptoms. Due to the newness of this type of therapy, long-term data is limited, but for one of the described techniques, published data show that the positive effects persist over a 2-year period.

**Summary:**

Transcatheter approaches are safe and are able to treat tricuspid regurgitation effectively. There are still differences in the efficacy of the different techniques. Clinical experience varies among the different approaches.

## Introduction

**Fig. 1:**
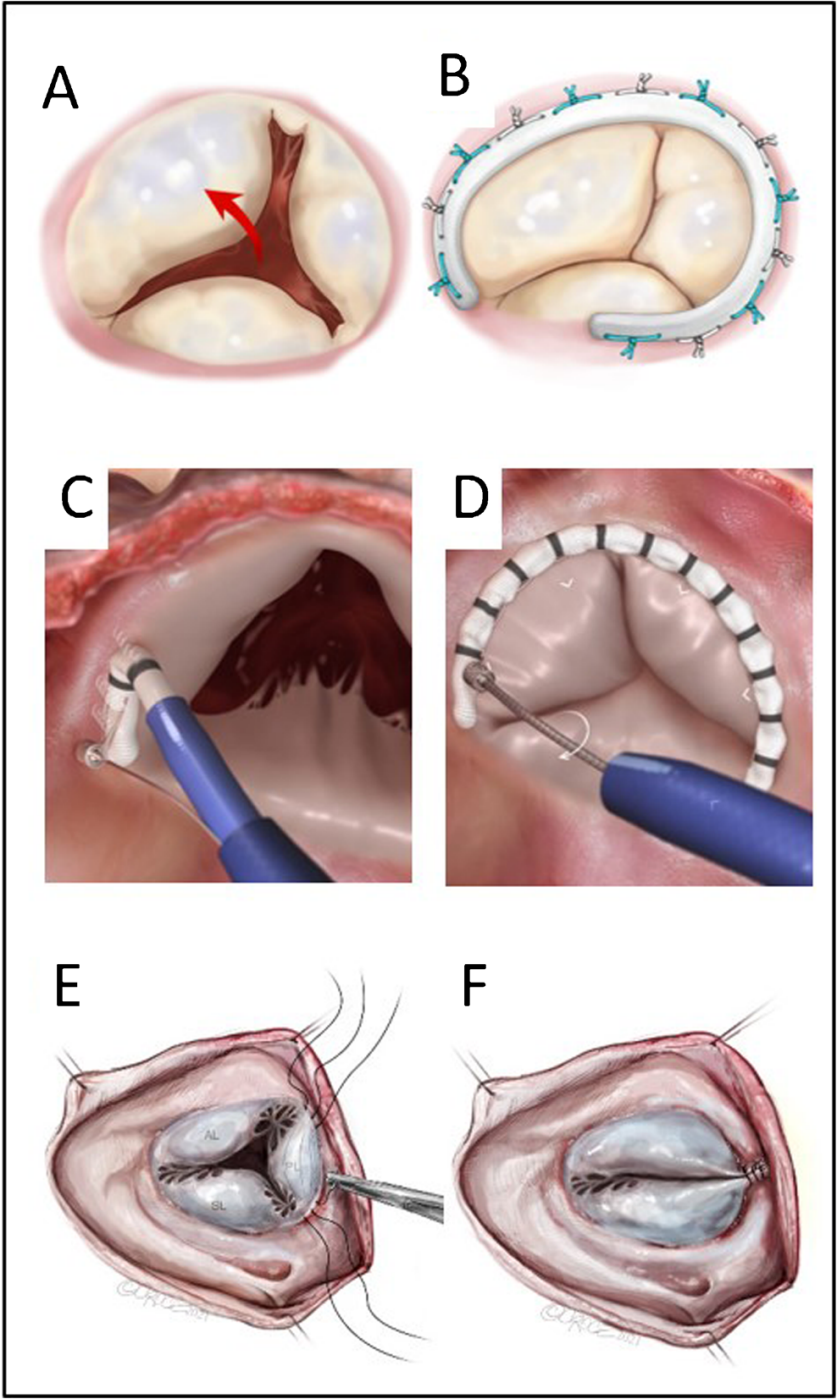
Overview of different annuloplasty techniques. **A** Functional TR with dilated tricuspid annulus and loss of coaptation of the tricuspid leaflets. **B** Restored leaflet coaptation after surgical annuloplasty with implantation of an annuloplasty ring. **C** Placement of anchors around the free wall of the tricuspid valve in order to attach the cardioband to the atrial aspect of the tricuspid annulus similar to a surgical implantation of an annuloplasty ring. **D** Contraction of the cardioband. **E** Technique of Kay’s procedure. Placement of sutures at the anterolateral and posteroseptal commissure of the tricuspid valve. **F** By tying down the sutures, the posterior leaflet is plicated, resulting in a bicuspidalized tricuspid valve. The TriAlign system and the TriCinch device mimic the Kay’s procedure. With the TriAlign system, a suture-based plication of the posterior leaflet is achieved via a transjugular access. The TriCinch device uses an anchor which is screwed into the anterolateral portion of the tricuspid annulus. It is pulled down toward the IVC on a Dacron band which is connected to a self-expanding stent. When tension is sufficient to reduce the degree of TR, the stent is released in the IVC (Figure **A**–**D** with permission from Edwards Lifesciences LLC, Irvine, CA. Edwards, Edwards Lifesciences, the stylized E logo, and its product names are trademarks of Edwards Lifesciences Corporation) (Figure **E**–**F** ©2021 Beth Croce)

Surgical techniques are extremely effective in reducing or elimination tricuspid regurgitation (TR). Preferably, annuloplasty techniques either suture-based or involving annuloplasty rings should be used for the treatment of functional TR instead of valve replacement. There is no class I indication for surgical treatment of isolated functional tricuspid regurgitation in the current guidelines [1, 2]. Intrahospital mortality after isolated tricuspid surgery is approximately 10%, and isolated tricuspid surgical procedures are infrequently performed [3]. This may be due to the fact that right heart failure and pulmonary hypertension are common conditions in these patients. All these comorbidities are known to be predictors of worse outcome [4]. Patients may therefore be referred late for surgical treatment or even not sent at all. In order to address this problem, several catheter-based techniques that mimic the surgical annuloplasty techniques have been developed. In this review, transcatheter approaches for tricuspid annuloplasty (Figure 1) that already have been introduced into the clinical arena (at a stage more than anecdotic case reports) are described and the related publications are summarized (Table 1).

**Table 1:** Overview of published clinical trials of different transcatheter annuloplasty techniques

Transcatheter annuloplasty system	Study title	Number of patients enrolled	Maximum duration of reported follow-up
Cardioband tricuspid valve reconstruction system	TRI-REPAIR [5, 6]	*n* = 30	2 years
	Early Feasibility Study US [7•]	*n* = 30	30 days
	German Real-World Registry [8]	*n* = 60	30 days
	TriBAND [personal communication]	*n* = 61	30 days
TriAlign	SCOUT [9]	*n* = 15	1 year
	SCOUT I and SCOUT II (pooled) [10]	*n* = 39	30 days
	TriValve [11]	*n* = 17	30 days
TriCinch	PREVENT [12]	*n* = 24	6 months
	TriValve [11]	*n* = 15	30 days

## Cardioband Tricuspid Valve Reconstruction System

The cardioband tricuspid valve reconstruction system (Edwards Lifesciences, Irvine, CA, USA) consists of a polyester fabric with incorporated contraction wire. Via a transfemoral venous access, it is affixed to the annulus with anchors. The number of anchors depends on the size of the cardioband. There are five sizes (A to F) available with the largest band requiring 17 anchors. Sizing is based on measurements derived from a cardiac CT scan. The implant catheter is steered to the atrial side of the tricuspid annulus close to the insertion of the leaflets starting close to the anterolateral commissure and continuing from the lateral to the posteroseptal part. The implantation is guided by transesophageal echocardiography (TEE) and fluoroscopy. A guidewire in the right coronary artery serves as landmark to facilitate orientation. The use of ICE can be helpful in cases with demanding anatomy [13]. In the final step, the band is contracted (up to maximum of 5.5 cm depending on the size of the cardioband) before it is released from the implant catheter. Contraction leads to a reduction of annular dimensions of the tricuspid valve allowing for better coaptation of the leaflets and reducing the degree of regurgitation.

The cardioband tricuspid valve reconstruction system received a CE mark in 2018. After the first-in-man procedure in 2016 [5], the TRI-REPAIR study included 30 patients who had been treated between 2016 and 2017 [6]. It was designed as a nonrandomized, single-arm, multicenter study which was conducted at 8 European centers. Inclusion criteria were symptomatic, chronic, functional tricuspid regurgitation (TR) with an annular diameter more than 40 mm. The patients were required to be on stable medication and deemed not suitable for surgery by the local heart teams. Exclusion criteria were LV-EF <30%, recent myocardial infarction, unstable angina, or recent coronary intervention, systolic pulmonary pressure > 60 mmHg, moderate or severe regurgitation or stenosis of other heart valves, chronic dialysis, anemia, cardiac cachexia, or life expectancy of less than 12 months. Currently, the follow-up up to 2 years was published [7•].

The primary endpoints were device success and the overall rate of major serious adverse events (MSAE). Device success was achieved in 100% of the patients. The rate of periprocedural MSAE was 13.3%. During the 30-day follow-up period, 2 deaths occurred. One patient died of right heart failure after occlusion of the right ventricular branch of the right coronary artery (RCA). Another patient died of intracranial bleeding which was not procedure-related. Other safety events were one stroke, four bleeding events, three coronary complications, and one patient with renal failure; one patient required permanent pacemaker implantation due to 3rd degree AV-block and two ventricular arrhythmias were detected. There were no incidents of myocardial infarction and no device-related cardiac surgery. The mortality rates were 10%, 16.7%, and 23.3% after 6, 12, and 24 months.

Cardioband implantation resulted in a reduction of anteroseptal diameter of the tricuspid annulus by 14%. This effect persisted over 12 months and 24 months. At baseline, 76% of the patients had a TR more than moderate (5 grade scale) evaluated by echocardiography, only 45% at discharge from the hospital and 37% and 27% at 12 month and 24 month, respectively. Patients experienced a significant improvement of their symptoms. At baseline, only 17% of the patients were in New York Heart Association (NYHA) class I or II but 78% at 1 year and 82% at 2 years. There was also a significant change with regard to quality of life as evaluated by KCCQ Score.

An early nonrandomized feasibility study [8] conducted at 9 sites in the USA reported the 30-day results of 30 patients. Patients were included if they had a symptomatic moderate or greater chronic functional TR and were determined as appropriate candidates for transcatheter tricuspid valve repair by the local heart team. Exclusion criteria were LV-EF <25%, severely depressed right ventricular function, previous surgery or device implantation on the tricuspid valve, pacemaker or ICD leads impinging the tricuspid leaflets, severely reduced kidney function, or anemia <9 mg/dl.

Device success was 93.3%. No patient died during the study. The anteroseptal diameter was reduced by 13%. In 85%, the TR could be reduced at least by 1 grade. In 44%, the severity of the TR was moderate or less. At 30 days, the NYHA functional class I or II in 75%. Seven bleeding events, one major access site problem, and one cardiac tamponade occurred. There were no myocardial infarctions, strokes, or conduction disorders.

In addition to the two abovementioned feasibility studies, data from a registry from 4 German centers represents initial “real-world” experience covering the early commercial use of the cardioband tricuspid valve reconstruction system [14]. Sixty patients with at least severe functional TR were treated. In the cohort were also patients with pacemaker or ICD leads, occluded RCA or proximity of the RCA to the tricuspid annulus who would have been excluded from the feasibility studies. The technical success rate was 96.7%. One patient was converted to open-heart surgery, because he developed a significant hematoma within the wall of the right ventricle during the procedure. In another patient, the device could not be contracted to the maximum. Nevertheless, the grade of the TR was reduced from severe to moderate in this case. Two patients died within 30 days after the procedure. Both deaths were not device-related. Further complications were symptomatic bradycardia with need for permanent pacemaker 3.3%, acute renal failure 11.7%, bleeding 11.7%, and cardiac tamponade 3.3%. In three patients, a perforation of the RCA occurred and 7 patients had a stent placed into the RCA. The rate of RCA interventions is of note. Acute deformation of the RCA is a frequent phenomenon after contraction of the cardioband. It was shown that the effect is transient [15••]. In the beginning of cardioband therapy, this was not known and may have led to overtreatment with stenting of the right coronary artery.

Reduction of the anteroseptal diameter was 24%. While at baseline in all of the patients, the severity of the TR was severe or more; 61% had an echocardiographic grade of their TR of mild or moderate at discharge. At 30 days, 81.2% were in NYHA functional class I or II. Compared to the two feasibility studies, the effect of the treatment is stronger. Less strict selection criteria with regard to the tricuspid valve anatomy in the registry may have contributed to these results.

The TriBAND study is an ongoing post market follow-up study. It is a single-arm non randomized study which is conducted in Europe. Up to now, 61 patients were enrolled.

## TriAlign

The TriAlign device (Mitralign Inc., Teksbury, MA, USA) is a suture-based system which allows to place two pledgets at a recommended distance of 2.4 to 2.8 cm on the posterior tricuspid leaflet. By suturing them together, the posterior leaflet is plicated resulting in a bicuspidalized tricuspid valve and reduction of annular dimensions. The device uses a transjugular access. The technique mimics the principles of Kay’s procedure, an established surgical technique for annuloplasty of the tricuspid valve [9].

The feasibility and safety was evaluated in the SCOUT trial [11]. A total of 15 patients were enrolled in 4 US centers. Patients were included when they had a chronic functional TR equal or greater than moderate and no indication for concomitant left-sided heart surgery. Exclusion criteria were age >85 years, pacemaker leads in place, sPAP >60 mmHg, LV-EF < 35%, and reduced RV function with TAPSE < 12 mm.

At 30 days, the device success was 80%. Three patients had a detachment of one of the pledgets. No deaths occurred. In one patient, the plication led to significant compromise of the blood flow in the RCA causing ST segment elevation which was successfully treated by stent implantation. In the intention-to-treat cohort, there was a significant reduction in TA diameter (4.0 ± 0.5 cm vs. 3.9 ± 0.5 cm, respectively) and in the as treated cohort, there was a significant reduction in mean vena contracta diameter (1.3 ± 0.4 vs. 1.0 ± 0.3, respectively; *p* < 0.022), TAA (12.3 ± 3.1 cm^2^ vs. 11.3 ± 2.7 cm^2^, respectively; *p* < 0.019), EROA by PISA (0.51 ± 0.18 cm^2^ vs. 0.32 ± 0.18 cm^2^, respectively; *p* < 0.020). Despite the relatively small changes of dimensions and echocardiographic parameters, there was a significant improvement in the NYHA functional class, 6-min walk test, and quality of life in the as treated cohort. In the TriValve registry, also 18 patients were included who had been treated with the TriAlign system [10]. The SCOUT II Study was planned as nonrandomized single-arm study which aimed to include 60 patients. First patient was enrolled in 2017. A pooled cohort from the SCOUT trial and initial patients from the SCOUT II trial was presented at the TCT meeting 2018 [16]. Device success after 30 days was 82%. Five patients experienced a detachment of one pledget. There was no need for reintervention. Nevertheless, the annular diameter was significantly reduced. NYHA functional class, 6-min walk test, and quality of life assessment were significantly better after treatment. Despite the above-described early clinical use of this device, it is not available anymore and no longer under development.

## TriCinch

Similar to the TriAlign system, the TriCinch system (4Tech Cardio Ltd., Galway, Ireland) is based on the principle of Kay’s procedure. The system consists of a corkscrew anchor, a self-expanding stent, and a Dacron band which connects the anchor to the stent. The screw is anchored to the anteroposterior annulus; then the stent is released in the hepatic region of the IVC. The Dacron band pulls the anchor toward the IVC thereby plicating the posterior leaflet [12]. In the Transcatheter Treatment of Tricuspid Valve Regurgitation with the TriCinch System (PREVENT) study, the feasibility and safety of the system was evaluated [17]. 24 patients were treated with the device. Device success was 81%. No patient died; 2 patients had pericardial effusions. Acute reduction of the TR severity by one grade was achieved in 94%. After 6 months, detachment of the anchor was found in 5 of 24 patients. In order to reduce the rate anchor detachments, a novel device was developed. It was anchored in the epicardial space [18]. In the meantime, the device is not available for clinical use anymore.

## Conclusion

Transcatheter techniques for tricuspid annuloplasty have shown to be safe and the results to be reproducible in the early clinical experience. Surprisingly already minor changes in tricuspid annular dimensions seem to translate into relevant improvement of symptoms and functional status of patients with functional tricuspid regurgitation. This might be attributed to the fact that by the heart teams involved, patients with a higher severity of the TR at a later stage of the disease were selected for the interventional treatment. This finding also led to the proposal of a new echocardiographic grading scale to describe the severity of TR [19]. In comparison to the two other described techniques, the cardioband tricuspid valve reconstruction system has the largest impact on tricuspid dimensions. No other device for transcatheter tricuspid annuloplasty has a higher number of patients or longer follow-up reported in clinical trials. From the three above-described systems, the cardioband tricuspid valve reconstruction system is currently the only one which is available for the clinical application. It is commercially available in Europe and an investigational device in the USA. Yet, clinical experience is still limited, and there is obviously room for further technical refinement and optimization of transcatheter annuloplasty systems. Several other transcatheter techniques for the treatment of tricuspid regurgitation besides annuloplasty are competing. In an analysis of the TriValve registry, lack of procedural success regardless of the repair technique was one of the strongest predictors of patient mortality [20•]. Correction of failed procedures with techniques that target the leaflets usually can only be achieved by open-heart surgery. In contrast, annuloplasty techniques leave the option of adding other transcatheter repair techniques during the initial procedure or later in the course or even the option of transcatheter valve replacement.

## Abbreviations

CE: Conformité Européenne
CT: computed tomography
EROA: effective regurgitant orifice area
ICD: implantable cardioverter defibrillator
ICE: intracardiac echocardiography
IVC: inferior vena cava
KCCQ: Kansas City Cardiomyopathy Questionnaire
LV-EF: left ventricular ejection fraction
MSAE: major serious adverse event
NYHA: New York Heart Association
PISA: proximal isovelocity surface area
RCA: right coronary artery
RV: right ventricle
sPAP: systolic pulmonary artery pressure
TAA: tricuspid annulus area
TAPSE: tricuspid annular plane systolic excursion
TCT: transcatheter cardiovascular therapeutics
TEE: transesophageal echocardiography
TR: tricuspid regurgitation
